# Symptoms and treatment when death is expected in dementia patients in long-term care facilities

**DOI:** 10.1186/1471-2318-14-99

**Published:** 2014-09-02

**Authors:** Maartje S Klapwijk, Monique AA Caljouw, Mirjam C van Soest-Poortvliet, Jenny T van der Steen, Wilco P Achterberg

**Affiliations:** 1 Department of Public Health and Primary Care, Leiden University Medical Center, P.O. Box 9600, 2300 RC Leiden, The Netherlands; 2 Marente, long-term care facility van Wijckerslooth, Oegstgeest, The Netherlands; 3 Department of General Practice & Elderly Care Medicine, EMGO Institute for Health and Care Research, VU University Medical Center, Amsterdam, The Netherlands

**Keywords:** Quality of dying, Long-term care, Symptoms, Dementia, End-of-life

## Abstract

**Background:**

Although dementia at the end of life is increasingly being studied, we lack prospective observational data on dying patients. In this study symptoms were observed in patients with dementia in the last days of life.

**Methods:**

When the elderly care physicians in two Dutch nursing homes expected death within one week, symptoms of (dis)comfort, pain and suffering were observed twice daily. For this the Pain Assessment in Advanced Dementia (PAINAD; range 0–10), Discomfort Scale-Dementia of Alzheimer Type (DS-DAT; range 0–27), End-Of-Life in Dementia-Comfort Assessment in Dying (EOLD-CAD; range 14–42) and an adapted version of the Mini-Suffering State Examination (MSSE; range 0–9), were used. Information on care, medical treatment and treatment decisions were also collected.

**Results:**

Twenty-four participants (median age 91 years; 23 females), were observed several times (mean of 4.3 observations (SD 2.6)), until they died. Most participants (n = 15) died from dehydration/cachexia and passed away quietly (n = 22). The mean PAINAD score was 1.0 (SD 1.7), DS-DAT 7.0 (SD 2.1), EOLD-CAD 35.1 (SD 1.7), and MSSE 2.0 (SD 1.7). All participants received morphine, six received antibiotics, and rehydration was prescribed once.

**Conclusion:**

In these patients with dementia and expected death, a low symptom burden was observed with validated instruments, also in dehydrated patients without aggressive treatment. A good death is possible, but might be enhanced if the symptom burden is regularly assessed with validated instruments. The use of observation tools may have influenced the physicians to make treatment decisions.

## Background

It is estimated that, worldwide, about 35 million people have dementia. Currently, 5% of people aged > 65 years are diagnosed with dementia, increasing to more than 50% in the group aged 90 years and over [1-3]. In the first stages of dementia people tend to live at home; however, when the disease becomes more progressive, many people with dementia are admitted to a long-term care facility (LTCF) [4]. In the Netherlands, and also in other parts of the world [5], LTCFs have specialized dementia care units. Daily medical care is provided by an elderly care physician specialized in care for vulnerable older people and the chronically ill living in a LTCF [4,6-8].

The period between ascertainment of the diagnosis dementia and death can take several years, with phases of slight to moderate decline or fast decline in cognition and functioning, depending on the type of dementia [9].

Internationally, many people with dementia die in LTCFs; e.g. in the USA 67% and in the Netherlands up to 92% [10]. The 6-month mortality rate in LTCF residents with advanced dementia is reported to be 18-37% [11,12].

The cause of death while dying with dementia has been studied in the USA and the Netherlands [13,14]. The most frequently mentioned cause of death is cachexia with dehydration (35%) [15]. Pneumonia and complications of cardiovascular disease were the second and third mentioned cause of death, respectively, both around 20% [15].

In the period before death patients can suffer from pain, dyspnea, agitation, anxiety, fear, crying, moaning, choking, gurgling or difficult swallowing. During this period, mouth care and the prevention of pressure ulcers, constipation and urinary retention are also important [16-18]. People with dementia are often incapable of expressing themselves verbally when they are uncomfortable or when they suffer from symptoms. Therefore, observational or proxy-rate instruments have been developed for people with serious cognitive impairment to measure the quality of dying; some of these instruments have good psychometric properties [19].

Differences in treatment during the final phase of patients with dementia have been acknowledged. In many countries (including the USA and some European countries) patients in the terminal phase too often receive aggressive treatments that may be of limited clinical benefit [14,20-24]. Especially the belief among many relatives and health-care workers about an unpleasant death when dehydrated, or the imagined effects of rehydration, may hamper a dignified and evidence-based palliative care [25,26].

Many studies have retrospectively described the symptoms in patients dying with dementia [14,18,20,27,28]. In these studies, data were collected retrospectively before and after death, to describe the experienced symptoms in the period before death. However, prospective observational studies that systematically observe dying patients with validated instruments are still lacking. Therefore, this prospective observational follow-up study was performed to describe the incidence and course of observed symptoms and treatment in people with dementia in the last days before their expected death.

## Methods

### Setting and study population

This prospective observational follow-up study was part of a study to validate methods of measurement of quality of care and quality of dying with dementia in long-term care facilities (LTCFs) in the Netherlands [19,29]. From January 2008 to February 2009 two elderly care physicians in two LTCFs included patients if they met the following inclusion criteria: residing in a LTCF for ≥ 30 days, a physician’s diagnosis of dementia and expected to die within the next 7 days. The expectancy of a patient to die within 7 days is based on an estimation made by the treating physician and the nurses caring for the patient, and is often related to the fact that a patient has stopped eating and drinking [30].

The Medical Ethics review Committee of VU University Medical Center Amsterdam approved the study. Families were asked for permission for study participation by the coordinating physician who also observed the patients. Neither of the observing elderly care physicians were part of the research group.

### Data collection

Two elderly care physicians collected data by filling out observation instruments, i.e. the Pain Assessment In Advanced Dementia (PAINAD), Discomfort Scale-Dementia Alzheimer type (DS-DAT), End-of-Life in Dementia scales-Comfort Assessment in Dying (EOLD-CAD), and the Mini Suffering State Examination (MSSE).

Prior to the start of this study, these two physicians were trained with an instructional video on the use of the DS-DAT and the PAINAD. The observations of the patients expected to die within 7 days were scheduled twice a day. During the observation periods, the patients were in rest. In the morning the PAINAD, the DS-DAT and the EOLD-CAD were scored. The physician in charge of the medical care observed the patient while sitting next to the patient for 10 minutes per observation.

The second observation was in the afternoon. Again, the physician observed the patient for 10 minutes and then filled out the PAINAD, DS-DAT and the MSSE, and additional questions regarding the course of the day. Therefore, the DS-DAT and PAINAD were scored twice a day (if possible), and the MSSE and EOLD-CAD once a day.

The PAINAD is an instrument that is validated to observe pain in non-communicative patients with advanced dementia [31]. The PAINAD contains 5 items which can generate a score from 0–2. The total score ranges from 0–10, with 10 indicating severe pain. A score of 2 or higher is used to give an indication of pain [32-34].

The DS-DAT measures discomfort in advanced dementia patients. It consists of 9 items with four response options ranging from 0–3. The total score ranges from 0–27, with 0 indicating ‘no discomfort’ and 27 indicating ‘the highest level of discomfort’ [35-37].

A tool to measure comfort at the end of life is the EOLD-CAD. This observation scale scores symptoms while dying with dementia and contains 14 items that can be scored with a 1, 2 or 3 score. The symptoms in the EOLD-CAD are the symptoms actually noticed at the time of the observation. The total score ranges from 14–42, with a higher score indicating a higher level of comfort for the patient [38,39].

Suffering was measured with the MSSE. The MSSE measures symptoms in end-stage dementia patients and gives an indication of suffering over the course of the whole day [40,41]. The MSSE has 10 items. One item is the family’s judgment regarding the suffering of the patient. Because data regarding suffering as seen by family members were retrospectively collected, these question in the MSSE was not used to calculate the total score; therefore, only the first 9 items were used for the present study. The total score ranges from 0 (indicating a low level of suffering) to 9 (indicating the highest level of suffering).

### Patient characteristics and treatment

Within two weeks after death, the physician collected information about the participants’ gender, age, marital status, length of stay at the LTCF, and duration of dementia. Information on care, medical treatment (including pain, and antipsychotic and anti-depressive medication), and treatment decisions in the last 7 days before death were also collected.

### Cognition

In addition, the 7 category Minimum Data Set Cognitive Performance Scale (CPS) was used to determine the cognitive performance status. The CPS was scored within 2 weeks after death and concerned the last month of life. The CPS is a valid measuring scale for cognitive performance. This scale can range from intact (level 0), borderline intact, mild, moderate, moderately severe and severe impairment to very severe impairment (level 6) [42]. Also, the 7-item Bedford Alzheimer Nursing Severity-Scale (BANS-S) was used to measure the severity of dementia in the last month before death. Scores on the BANS-S range from 7–28; a score of 17 and higher is regarded as severe dementia [43,44].

### Statistical analysis

Descriptive statistics were used to describe the study population and observed symptoms; results are reported as mean and standard deviation (SD) for normally distributed data, and median and interquartile range (IQR) for non-normally distributed data. The t-test was used to compare age, duration of stay at the LTCF and years of dementia between the observed and non-observed patients.

To dichotomize the presence of symptoms in the EOLD-CAD the scores 1 = ‘a lot’ and 2 = ‘somewhat’ are combined.

Descriptive statistics reported the mean and SD of the observational instruments at each observation point before death.

All analyses were performed with SPSS statistical software, version 20 (SPSS Inc., IBM, USA).

## Results

### Study population

During the study period from February 2008 to February 2009 in two Dutch LTCFs, a total of 36 patients died on the wards in which the physicians were working. Of these, 12 patients could not be included in the present study because of sudden death (n = 5) or because the physician did not have the opportunity to perform the observations (n = 7), resulting in 24 participants available for this study. Of these participants, 11 were observed only one time and 13 were observed more frequently; the mean number of observations was 4.3 (SD 2.6). In total, 80 observations were conducted by the two physicians. All 24 participants died within 5 days; 12 of them died within the first 2 days.

Of the 24 observed participants (23 females) the mean age was 90 (SD 6.9) years. Mean length of stay in the LTCF was 32 (SD 27.8) months. The mean duration of dementia was 49 (SD 41.7) months. No difference in age and length of stay was found between the 24 participants and the 12 non-participants.

### Cognition and severity of dementia before dying

About half of the participants (52.4%) had very severe cognitive impairment. The mean BANS-S score was 19 (SD 5.4) (Table 1).

**Table 1:** Baseline characteristics of the study population (N = 24) and course of mortality

***Socio-demographic factors***		
Female, n (%)	23	(95.8)
Dutch, n (%)	23	(95.8)
Widowed, n (%)	16	(66.7)
Mean age in years (SD)	90	(6.9)
Mean length of stay in months (SD)	32	(27.8)
***Medical information***		
Dementia mean duration in months (SD)	49	(41.7)
***Cognition***		
CPS		
Level 0 Intact, n (%)	0	(0)
Level 1 Borderline intact, n (%)	1	(4.2)
Level 2 Mild impairment, n (%)	0	(0)
Level 3 Moderate impairment, n (%)	1	(4.2)
Level 4 Moderate severe impairment, n (%)	0	(0)
Level 5 Severe impairment, n (%)	9	(37.5)
Level 6 Very severe impairment, n (%)	13	(54.2)
BANS-S mean score (SD)	19	(5.4)

SD = Standard deviation.

CPS = Cognitive Performance Scale.

BANS-S = Bedford Alzheimer Nursing Severity-Scale.

### Symptoms of dying

**Figure 1:**
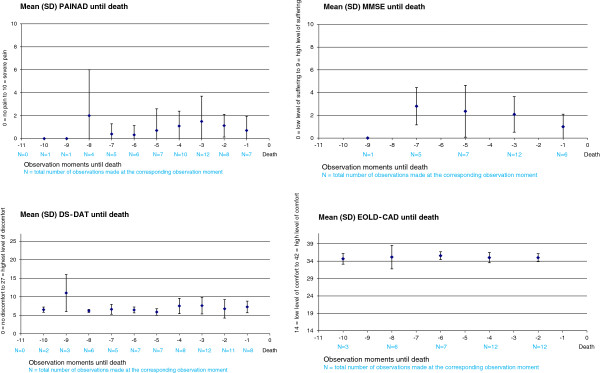
Mean score of observational instruments (with standard deviation) in last days to death.

The PAINAD was completed 61 times (missing 19 times); for 39 of these ratings (63.9%) no indication of pain was observed. There were 69 DS-DAT ratings (11 missing), 31 MSSE ratings (9 missing) and 40 EOLD-CAD ratings.The mean PAINAD score was 1.0 (SD 1.7), the mean DS-DAT score was 7.0 (SD 2.1), the mean EOLD-CAD score was 35.1 (SD 1.7), and the mean MSSE score was 2.0 (SD 1.7). Figure 1 shows the course of the total scale scores until death, which overall implies a low prevalence of symptoms.

The scores of the PAINAD for each participant resulted in a total of 6 participants (26%) that always scored zero (no indication of pain). All 24 participants scored one or more points on the DS-DAT. Two participants (10%) scored no symptoms of suffering on the MSSE.

Table 2 shows the symptoms of dying scored with the MSSE and EOLD-CAD in the 7 days before death. The MSSE was conducted 31 times, 7 of these ratings (22.6%) had a score of 0, and 24 ratings (77.4%) had a score of 1–9, indicating some symptoms of suffering. In multiple observations, symptoms such as pain were present 11 times (35.5%), malnutrition was present 13 times (41.9%), eating disorders 12 times (38.7%) and suffering according to medical opinion 10 times (32.3%).

**Table 2:** Symptoms present in multiple observations in ≤ 7 days before death in all 24 patients

	**N**	**%**
**MSSE**	31	100
Restlessness/not calm	6	19.4
Screams	2	6.5
Pain	11	35.5
Decubitus ulcers	2	6.5
Malnutrition	13	41.9
Eating disorders	12	38.7
Invasive action	1	3.2
Unstable medical condition	4	12.9
Suffering according to medical opinion	10	32.3
**EOLD-CAD**	40	100
Discomfort	15	37.5
Pain	6	15.0
Restlessness/not calm	7	17.5
Shortness of breath	12	30.0
Choking	4	10.0
Gurgling	2	5.0
Difficulty swallowing	8	20.0
Fear	2	5.0
Anxiety	5	12.5
Crying	0	0
Moaning	6	15.0
Serenity	34	85.0
Peace	36	90.0
Calm	35	87.5

MSSE = Mini-Suffering State Examination; missing n = 9.

EOLD-CAD = End-Of-Life in Dementia-Comfort Assessment in Dying.

For example, the observational instrument EOLD-CAD scored discomfort among participants 15 times (37.5%), shortness of breath 12 times (30%), serenity 34 times (85%), peace 36 times (90%), and calmness 35 times (87.5%).

### Treatment and medication

In the last days before death, 12 of the participants lost consciousness. According to the physicians, 22 participants (91.7%) passed away quietly in the last 6 hours before death and 2 (8.3%) were aware of symptoms.

All 24 participants received morphine (one received a dose that was higher than necessary for symptom control); for 19 participants (79,1%) it was not necessary to increase the dose of morphine in the course of the terminal phase. In 4 patients (16.7%) there was a gradual increase in the dosage of morphine and, in one patient (4.2%), there was a substantial increase in the dosage on the last day. Two participants were pharmacologically kept sedated, in order to relieve the symptom burden. The exact dosages of paracetamol, NSAIDs, anti-depressive and antipsychotic medication, were not available for all patients.

Six participants (25%) received antibiotics and one (4.2%) received subcutaneous rehydration. The physicians reported that they stopped antibiotics or oral medication or rehydration in 13 (54.2%) of the participants, for one participant (4.2%) they decided not to start antibiotics, two participants were not sent to the hospital for surgery, and for one participant no further diagnostic exploration was started (Table 3).

**Table 3:** Medication/palliative care and cause of death ≤ 7 days before death in the study population (N = 24)

	**N**	**%**
***Medication/palliative care***		
Antibiotics, oral tablets	6	25.0
Morphine	24	100
Rehydration, hypodermoclyse	1	4.2
Stop antibiotics, oral medication or rehydration	13	54.2
Not starting treatment with antibiotics	1	4.2
Not starting treatment with surgical operation	2	8.3
Not starting further exploration in diagnosis	1	4.2
Missing	3	12.5
***Cause of death***		
Cachexia/dehydration	15	62.5
Pneumonia (acute pulmonary disease)	3	12.5
Disease of the digestive system	4	16.7
Renal failure	1	4.2
Brain injury after a fall	1	4.2

The physicians also reported that no treatment was stopped or withheld to induce death.

Five participants had an indwelling urinary catheter and two received oxygen. All participants received mouth care, and 19 treatments for the prevention of pressure ulcers were conducted.

### Cause of death

Fifteen participants (62.5%) died of cachexia/dehydration, three of pneumonia (12.5%) and four (16.7%) due to a disease of the digestive system, one from renal failure and one died from brain injury after a fall (4.2%) (Table 3).

## Discussion

This is one of the few studies that prospectively and through direct physician observation explored the symptomatology and treatment in the last days of life of patients with advanced dementia.

The low level of burdensome symptoms in the days before death, also in patients dying from dehydration/cachexia, the low rate of aggressive curative treatment and the high rate of palliative treatment with morphine are striking results. These data strengthen the recommendations for a better tailored, less curative aggressive approach to palliative care in dementia [24,45].

A strength of this study is the prospective study design. Retrospective designs in end of life care are particularly sensitive for bias [46]. Particularly when people are easily identified to be at risk of dying (which was the case in this study), this prospective approach is recommended [47].

Another strength is that this study used structured observations twice a day (with validated instruments) by physicians specialised in the care for dementia patients [19,48]. The results of the total scores of the observational instruments showed no increase in symptom burden over time, not even on the last days prior to death. This is a remarkable contrast to the results seen in other retrospective studies, which showed an increase in burdensome symptoms in the time prior to death [14,49].

This study also has some limitations. It was a relatively small sample, there were missing observations, and the observations were performed by the elderly care physicians who also were responsible for the treatment decisions. Therefore, an important issue to consider is whether these findings have been influenced by the study itself. Having a physician who performs a structured observation of symptoms twice a day, might lead to an improved awareness and assessment of symptomatology and, hence, to better palliative treatment.

Studying symptoms of dying in patients with dementia is challenging. Although differentiation between pain and, for instance, anxiety or discomfort is difficult, in the present study specific observational instruments were used. The DS-DAT was specifically developed to measure discomfort in advanced dementia, and the PAINAD was developed and validated for the assessment of pain in people with dementia [31-37]. The PAINAD has not been validated for use in the last days of life. The item ‘breathing’ in the PAINAD covers ‘noisy labored breathing’ , ‘long periods of hyperventilation’ or ‘Cheyne-Stokes respirations’. Cheyne-Stokes respirations are often seen in the last days of life and can be part of the normal physiological process of dying. However, because we cannot differentiate between the three symptoms observed under ‘breathing’ , we cannot state exactly how many people exhibited Cheyne-Stokes respirations.

The EOLD-CAD includes gurgling/death rattle but does not include, for instance, nausea or vomiting, two symptoms that can also be unpleasant in the last days of life. Also, the presence of a death rattle cannot differentiate between the underlying cause, for instance pneumonia or the absence of coughing. The best treatment for death rattle in dementia is morphine or an anticholinergic drug, like scopolamine. However, more studies are needed to determine the most effective treatment. It would be interesting to repeat the present study and include independent, objective observers and also include observations after a change in treatment. At the time of this study, the validated instruments used were the best available. These observational instruments help to structurally assess symptoms in these patients, and can help to observe symptoms and to assess the effect of the treatment received for these symptoms.

All patients received morphine and, in 19 (79.1%) of these patients, the dosage was not raised. We do not want to imply that the use of morphine in the last days of life with dementia is always necessary, but it is often used to alleviate the burden of pain or dyspnea. In the Netherlands, many patients receive the starting dose of morphine (10–30 mg subcutaneously a day), for a considerable period of time (days, weeks, sometimes even months). Death is not considered to be a direct side-effect of this medication in these dosages, and (as far as we know) there is no evidence for any association between this treatment and death.

Elderly care physicians in the Netherlands are in charge of the medical care of patients in the long-term care facility [8]. They have received extensive training in elderly care medicine, which includes palliative care. In this study we decided to observe the patients when death was expected within 7 days. The moment of death can be more accurately predicted when the intake of fluid or food has severely diminished; in the present study, the result was that 50% of the patients died within 2 days after the start of the observation. The cause of death was assessed by the elderly care physicians who were fully knowledgeable about the patient’s condition in the last phase of life.

In the discussion about a good death, physician involvement such as physician-assisted suicide, euthanasia, palliative sedation and withholding curative treatment are heavily debated. In 2010, of all deaths in the Netherlands, 2.8% were the result of euthanasia, and 12.3% of the deaths were the result of continuous deep sedation until death [50]. In a study covering the period 2007 to 2011 in dementia patients, 21% received deep sedation [18]. However, euthanasia is very rare in patients with dementia (although under Dutch law dementia is not an exclusion criterion *per se*) and in the present study there were no such cases. Also, for none of the patients in the present study, was starting or withholding treatment performed with the intention to induce death. This is in line with the observation that, in the Netherlands, active physician involvement in inducing death in dementia patients in long-term care is extremely rare [50]. Therefore, active physician involvement in this setting seems to mainly consist of pertinent palliative care principles.

## Conclusion

In this study in patients with advanced dementia and expected death, a low symptom burden was observed with validated instruments, also in dehydrated patients without aggressive treatment. A good death is possible, but this might be enhanced in a situation where symptom burden is regularly assessed with validated instruments. Therefore, we support others who recommend that proper symptom assessment should be implemented in long-term care. The use of observation tools may help physicians to take appropriate treatment decisions. All symptoms and preventive measures should feed into a personalised tailored care plan to help the patient and the (in)formal caregivers in the process of dying with dementia.

## Competing interests

The authors declare that they have no competing interests.

## Authors’ contributions

MCvSP and JTvdS had full access to all data in the original study and take responsibility for the integrity of the data. MSK, MAAC, MCvSP, JTvdS and WPA contributed to the study concept and design, analysis and interpretation of the data, drafting and critical revision of the manuscript. All authors have read and approved the final version of the manuscript.

## Pre-publication history

The pre-publication history for this paper can be accessed here:

http://www.biomedcentral.com/1471-2318/14/99/prepub
